# Comparative genomics of *Vibrio campbellii* strains and core species of the *Vibrio* Harveyi clade

**DOI:** 10.1038/srep41394

**Published:** 2017-02-01

**Authors:** Huei-Mien Ke, Anuphap Prachumwat, Chun-Ping Yu, Yi-Ting Yang, Sutitcha Promsri, Kuan-Fu Liu, Chu-Fang Lo, Mei-Yeh Jade Lu, Mei-Chin Lai, Isheng J. Tsai, Wen-Hsiung Li

**Affiliations:** 1 Ph.D. Program in Microbial Genomics, National Chung Hsing University and Academia Sinica, Taiwan; 2 Biodiversity Research Center, Academia Sinica, Taipei, Taiwan; 3 Centex Shrimp, Faculty of Science, Mahidol University, Bangkok, Thailand; 4 Shrimp-Virus Interaction Laboratory, Animal Biotechnology Research Unit, National Center for Genetic Engineering and Biotechnology (BIOTEC), National Science and Technology Development Agency (NSTDA), Pathum Thani, Thailand; 5 Institute of Bioinformatics and Biosignal Transduction, College of Bioscience and Biotechnology, National Cheng Kung University, Tainan, Taiwan; 6 Department of Life Science, National Taiwan University, Taipei, Taiwan; 7 Department of Biotechnology, Faculty of Science, Mahidol University, Bangkok, Thailand; 8 Tungkang Biotechnology Research Center, Fisheries Research Institute, Council of Agriculture, Pingtung, Taiwan; 9 Center of Bioscience and Biotechnology, National Cheng Kung University, Tainan, Taiwan; 10 Department of Life Sciences, National Chung Hsing University, Taichung, Taiwan; 11 Agricultural Biotechnology Center, National Chung Hsing University, Taichung, Taiwan; 12 Department of Ecology and Evolution, University of Chicago, Chicago, US

## Abstract

The core of the *Vibrio* Harveyi clade contains *V. harveyi, V. campbellii, V. owensii, V. jasicida*, and *V. rotiferianus*. They are well recognized aquatic animal pathogens, but misclassification has been common due to similarities in their rDNA sequences and phenotypes. To better understand their evolutionary relationships and functional features, we sequenced a shrimp pathogen strain *V. harveyi* 1114GL, reclassified it as *V. campbellii* and compared this and 47 other sequenced *Vibrio* genomes in the Harveryi clade. A phylogeny based on 1,775 genes revealed that both *V. owensii* and *V. jasicida* were closer to *V. campbellii* than to *V. harveyi* and that *V. campbellii* strains can be divided into two distinct groups. Species-specific genes such as intimin and iron acquisition genes were identified in *V. campbellii*. In particular, the 1114GL strain contains two bacterial immunoglobulin-like genes for cell adhesion with 22 Big_2 domains that have been extensively reshuffled and are by far the most expanded among all species surveyed in this study. The 1114GL strain differed from ATCC BAA-1116 by ~9% at the synonymous sites, indicating high diversity within *V. campbellii*. Our study revealed the characteristics of *V. campbellii* in the Harveyi clade and the genetic basis for their wide-spread pathogenicity.

The genus *Vibrio* is a bacterial group widely distributed in the marine environment. The core of the *Vibrio* Harveyi clade consist of *V. harveyi* and its closely related species *V. campbellii, V. owensii, V. jasicida* and *V. rotiferianus*^1^,^2^,^3^, all of which are well recognized aquatic animal pathogens^4^,^5^,^6^,^7^,^8^,^9^,^10^. Their members are commonly used as models to study bacterial luminescence^11^,^12^,^13^, quorum sensing^14^, biofilm formation, multi-chromosomal genome organization^15^,^16^, and recombination patterns^2^. Their genetic and phenotypic signatures are highly similar; conventional biochemical tests and 16 S rDNA sequencing frequently led to species misidentifications in this clade^17^,^18^. A well-known example is the misclassification of *V. campbellii* as *V. harveyi* – while the two species share only 61–74% DNA sequence similarity, the similarity in their 16 S rDNA sequences is over 97%^1^,^17^. Consequently, multi-locus sequence analysis (MLSA) was later used for species classification^1^,^18^,^19^,^20^. Given the sequence of two concatenated house-keeping gene sets (*ftsZ, mreB* and *topA*, or *rpoD, rctB* and *toxR*), the Harveyi clade ATCC BAA-1116 and HY01 were found to be *V. campbellii* rather than *V. harveyi*^18^.

The advent of next-generation sequencing has enabled multiple genome sequencing and assembly of *Vibrio* species. Such data have aided the study of phylogenomics^2^,^3^,^21^ and the identification of diagnostic features in *Vibrio* species^22^. A recent study of *V. harveyi* compared the genome of this species with those of three other related ones^21^. Nevertheless, a detailed genomic analysis of the frequently misidentified pathogen *V. campbellii* is still absent. High quality genomes of *Vibrio* species are difficult to obtain because of the high copy number rRNA operon present in the two chromosomes of *Vibrio* species (8–12). In the core Harveyi clade, to date, only the genomes of *V. campbellii* ATCC BAA-1116, *V. harveyi* ATCC 43516, and *V. harveyi* ATCC 33843 have been assembled into chromosomes. The other published genome assemblies remained fragmented, with 42 to 6,533 scaffolds (last assessed: 2015.09.17).

By applying genome-wide data, microbial taxonomy can be established via (1) average nucleotide identity (ANI) among sequences between two genomes^23^, and (2) the phylogeny of concatenated single copy orthologues, such as a core genome tree^2^,^21^. ANI ≥ 95% against type strains has become a commonly accepted definition of species^3^,^24^. Additionally, the combination of species assignment based on genomic distance and the monophyletic groupings allow clear species delineation^2^,^3^,^25^. Once the correct taxonomy is produced, shared genomic features and phenotypes of strains in a monophyletic group of interest can be established.

We had three goals in the current study. First, we sequenced the genome of *V. harveyi* 1114GL, which is a virulent strain isolated from a black tiger shrimp-culturing pond in Thailand in 2005^26^,^27^. Second, we compared this new genome with 47 other published genome assemblies to identify species-specific differences in gene content and protein domains. Lastly, we established a reliable Harveyi clade phylogeny to reduce potential misclassification of species or strains in this clade.

## Results and Discussion

### Genome sequencing of *V. harveyi* 1114GL and re-classification of it as *V. campbellii*

*V. harveyi* 1114GL is the main cause of shrimp deaths in Thailand^26^,^27^,^28^, and thus, it attracted our interest to sequence its genome. The genome was sequenced using both Illumina and Roche 454 technologies (see Methods) and assembled into two scaffolds corresponding to chromosomes I (3,515,540 bp with 6 gaps) and II (2,118,471 bp with no gaps). The ANI between 1114GL and the type strain *V. campbellii* NBRC 15631 was 96.3%. Based on the threshold of 95–96% ANI for the same species, 1114GL was reclassified as *V. campbellii*. This is the most well assembled *V. campbellii* genome assembly after the genome assembly of ATCC BAA-1116. Among the 4,991 proteins predicted by PROKKA^29^, only 12 (0.24%) did not have significant matches in the nr database. There was no plasmid sequence found in the 1114GL assembly, an observation consistent with the negative result of plasmid extraction from 1114GL in a previous study^27^.

### Phylogeny and genome content for a highly diverse set of *Vibrio* strains

**Figure 1:**
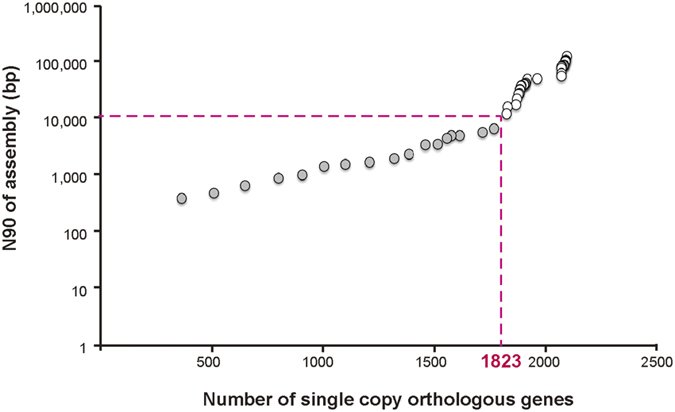
Number of single copy orthologous genes inferred by OrthoMCL using assembly N90. The starting point includes 19 strains: *V. campbellii* 1114GL, ATCC BAA-1116, NBRC15631, UMTGB204, and HY01; *V. owensii* 47666-1, DY05, and 1DA3; *V. jasicida* BSW5 and BSW7; *V. harveyi* CAIM1792, VH5, and VH2; *V. parahaemolyticus* O3:K6 substr. RIMD 2210633, BB22OP, O1:Kuk str. FDA_R31, O1:K33 str. CDC_K4557, UCM-V493, and FORC_008. The N90 of these 19 genomes ranged from 13,457 to 2,195,939 bp. The number of single copy orthologous genes was found to decrease sharply when strains with N90 < 10 kb assemblies were included. To increase annotation quality, only the genomes with N90 > 10 kb were chosen for further analysis in this study. The final dataset consisted of 48 strains (12 from *V. campbellii,* 15 from *V. harveyi,* 7 from *V. owensii,* 5 from *V. jasicida,* 3 *V. rotiferianus*, and 6 from *V. parahaemolyticus*) and they shared 1,823 single copy orthologs.

**Figure 2:**
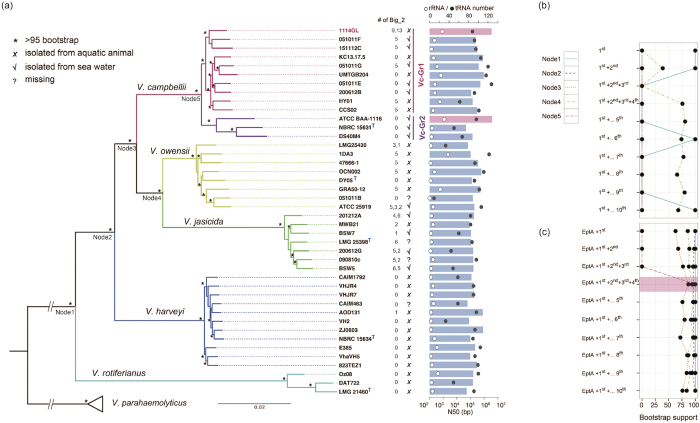
Phylogenic tree of the core members of the Harveyi clade. (**a**) A total of 1,775 amino acid sequences from single copy orthologous genes were used to construct a maximum likelihood phylogeny tree, which separates the core clade into 5 groups. The strain names are indicated in each branch of the tree. The copy number of Big_2 domain, isolation source, N50 of the genome assembly (bar chart), and the copy numbers of rRNA (open circle) and tRNA (solid circle) are given on the right hand side of the figure. (**b**) The bootstrap values in the phylogenetic trees reconstructed using the proteins selected from the correlations between sequence similarities and branch length of the phylogenetic tree from (**a**). Node1 to Node5 of the trees correspond to the nodes of the phylogenetic tree in (**a**). The top ten proteins are: (1) hypothetical protein, (2) 1-deoxy-D-xylulose 5-phosphate reductoisomerase, (3) glutamate-cysteine ligase, (4) lipoprotein, (5) N-acetylglucosamine-6-phosphate deacetylase, (6) D-cysteine desulfhydrase, (7) NAD dependent epimerase/dehydratase family protein, (8) Peptidoglycan hydrolase FlgJ, (9) muropeptide transporter, and (10) Flagellar hook-associated protein 3. (**c**) The bootstrap values from the phylogentic trees reconstructed by concatenating phosphoethanolamine transferase (EptA) and the top one to top ten genes that were added serially. The tree reconstructed by concatenating EptA and the top four proteins correctly classified both the species and the two *V. campbellii* groups with strong bootstrap support.

To infer an accurate phylogeny of available strains in the core Harveyi clade, we initially collected all 59 available genomes for the species in the core Harveyi clade and 6 additional genomes from *V. parahaemolyticus* as the outgroup (see Methods; Supplementary Table S1; Supplementary Table S2). However, only 362 single copy orthologous genes across all the species were identified, which is much lower than the 897 across 35 *Vibrio* strains of the Harveyi clade observed by Urbanczyk *et al*.^2^. This might be due to the lower annotation quality of the fragmented assemblies. Indeed, the number of single copy orthologous genes was found to decrease sharply when strains with poorer assemblies were included (Fig. 1). When assemblies with N90 < 10 k bp were filtered out, 1,775 single copy orthologous proteins (on average 34.8% of the proteome) were identified among 48 strains (12 from *V. campbellii,* 15 from *V. harveyi,* 7 from *V. owensii,* 5 from *V. jasicida,* 3 from *V. rotiferianus*, and 6 *from V. parahaemolyticus*) (see Supplementary Table S2). A concatenated alignment from these proteins was used to construct a maximum likelihood phylogeny tree with strong bootstrap clearly separating different species in the Harveyi clade and the *V. parahaemolyticus* outgroup (Fig. 2a).

The phylogenetic tree revealed inconsistencies in the species classification. Four strains in the phylogeny (1114GL, KC13.17.5, BSW7 and BSW5) were previously identified as *V. harveyi*^30^,^31^. Here we reclassified 1114GL and KC13.17.5 as *V. campbellii* strains, and BSW7 and BSW5 as *V. jasicida* strains. Their ANI values showed congruent results with this designation (see Supplementary Table S3): ANI was 96.3% between KC13.17.5 and the type strain *V. campbellii* NBRC 15631, 97.9% between BSW5 and its type strain *V. jasicida* LMG25398, and 97.9% between BSW7 and the type strain *V. jasicida* LMG25398. An interesting observation is that in the phylogeny *V. owensii* and *V. jasicida* were closer to *V. campbellii* than to *V. harveyi,* different from that of a previous study using only 138 genes^3^.

*V. campbellii* strains can be classified into two major groups, denoted as Vc-Gr1 and Vc-Gr2 (Fig. 2a). The separation of the two groups in *V. campbellii* had also been suggested by two previous studies, using concatenated sequences of 897^2^ or 1,615^21^ protein-coding genes.

We sought to identify a minimum number of key genes to reproduce the same classification based on calculations of correlations between phylogenetic topology from amino acid sequence and DNA sequence similarities of orthologous genes (see Methods). Briefly, we ranked the single copy orthologues by correlations and reconstructed the phylogeny (see Supplementary Table S4). The first gene (Pearson’s r = 0.99) was able to separate *V. rotiferianus* from the other species of the core Harveyi clade (Fig. 2b). Adding the second gene resulted in the correct classification of all the species. The first and second genes encode a hypothetical protein and 1-deoxy-D-xylulose 5-phosphate reductoisomerase, respectively. Adding more genes did not separate the two *V. campbellii* groups; hence we produced an alternative list which was ranked according to the topology of the *V. campbellii* subtree. Using the top gene from this new list (Pearson’s correlation coefficient = 0.960), the gene for a phosphoethanolamine transferase (EptA), and two more genes (for glutamate cysteine ligase and lipoprotein), which are from the old list, correctly classified both the species and the two *V. campbellii* groups with strong bootstrap support (Fig. 2b). The EptA is involved in the addition of phosphoethanolamine to lipid A and is required for polymyxin resistance^32^. It will be interesting to study the protein diversity in these species and the two groups of *V. campbellii*.

### *V. campbellii-*specific features common to all strains

We sought to identify genes specific to *V. campbellii*. As some of the published genomes used in this study are fragmented, we focused on protein domains, which are modules of protein structure^33^, so that they are robust against potential misannotations such as gene fusion. Among the 313 enriched Pfam domains in *V. campbellii* by Wilcoxon rank-sum test (p ≤ 0.05) (see Supplementary Table S5a), 51 were present only in *V. campbellii* strains. Five of these domains in six proteins of ATCC BAA-1116 were found in all *V. campbellii* strains. The product descriptions and IDs of these genes in ATCC BAA-1116 and 1114GL are shown in Supplementary Table S5b. Three of these proteins are related to bacterial fitness: the rhizobactin siderophore biosynthesis protein with K_oxygenase domain which increases the ability of iron aquisition^34^, the putative neutral zinc metallopeptidase with Zn_peptidase domain known to increase fitness by cleaving proteins in *Vibrio*^35^, and the hypothetical protein with IAT_beta found in intimins and invasins, which are adhesin and virulence factors produced by gram-negative bacteria^36^. These *V. campbellii -*specific genes may be used as characteristics for distinguishing *V. campbellii* from the other members of the core Harveyi clade. The 313 expanded domains were enriched in GO terms related to bacterial flagellum formation including “bacterial-type flagellum-dependent cell motility”, “bacterial-type flagellum assembly”, and “bacterial-type flagellum organization”, as well as virulence-related GO terms such as “protein secretion by the type III secretion system”, “chitin catabolic process”, and “proteolysis”^37^,^38^ (see Supplementary Table S6).

However, *V. campbellii* also appears to have lost four domains that all other members of the core Harveyi clade and *V. parahaemolyticus* contained at least one copy of them. They are Glucodextran_C (C-terminal binding-module, SLH-like, of glucodextranase), Omp_AT (solitary outer membrane autotransporter beta-barrel domain), ydhR (putative mono-oxygenase ydhR), and YHS (found in copper transporting ATPases, some phenol hydroxylases and uncharacterized membrane proteins) (see Supplementary Table S5c).

### Identification of determinants associated with *V. campbellii* Group 1 or 2

Our virulent isolate 1114GL was clustered in Group 1 (Vc-Gr1) with the known shrimp pathogen HY01^39^; half of the strains in this group were also associated with aquatic animals with strong bootstrap support (Fig. 2a). Group 2 (Vc-Gr2) included the published complete genome ATCC BAA-1116 and other strains isolated from seawater. There is no apparent ecological factor contributing to the separation of these two groups: only half of the Vc-Gr1 members were isolated from aquatic animals and all three Vc-Gr2 members were isolated from seawater. Next, we investigated the copy number differences in Pfam domains between these two groups (p ≤ 0.05; Wilcoxon rank-sum test). The phylogenetic position and the patterns of the expanded/lost domains of Vc-Gr1 were closer to the other four *Vibrio* species than to Vc-Gr2 (Fig. 2a, Supplementary Fig. S1, Table S5d, and Table S5e), suggesting that the domains enriched in Vc-Gr1 were likely present in their ancestors and were subsequently lost in Vc-Gr2.

**Figure 3:**
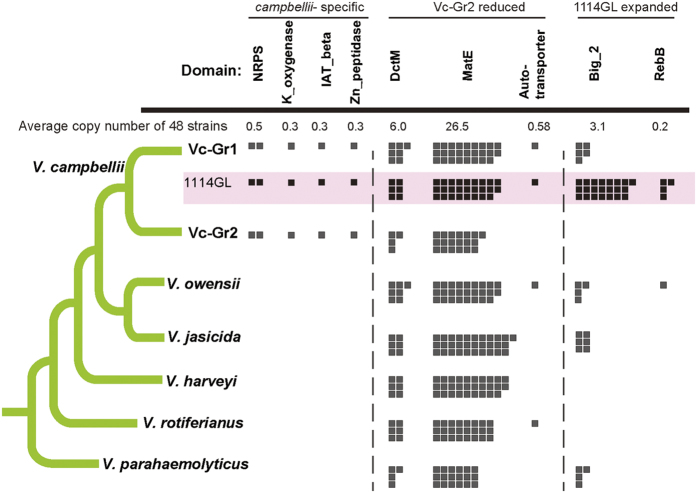
Summary of expanded domain families in *V. campbellii*, Vc-Gr1, or 1114GL. The number of squares displays the average number of domains in a genome from a cluster. The copy numbers for Vc-Gr1 include 1114GL. The numbers from each strain are shown in Supplementary Table S5.

A total of 386 Pfam domains were significantly expanded in Vc-Gr1 (see Supplementary Fig. S1a and Table S5d) and were enriched in the functions related to ‘antibiotic transport’ and ‘galactose metabolic process’ (Supplementary Table S7a). For example, there were on average 25.9 copies (20–29 copies) of the MatE domain (Multi antimicrobial extrusion protein) in Vc-Gr1, but only 18.7 copies in Vc-Gr2 (17–20 copies) (Fig. 3). In ATCC BAA-1116, there are ten proteins with two MatE domains (AGU93723.1, AGU93934.1, AGU94255.1, AGU95063.1, AGU95118.1, AGU95453.1, AGU95978.1, AGU97030.1, AGU97571.1, and AGU97766.1). In 1114GL, ten proteins (Vca1114GL_00245, Vca1114GL_00475, Vca1114GL_00731, Vca1114GL_01057, Vca1114GL_02103, Vca1114GL_02186, Vca1114GL_02598, Vca1114GL_03288, Vca1114GL_04301, and Vca1114GL_04662) were identified using OrthoMCL^40^ to be orthologous with the ten proteins in ATCC BAA-1116. In addition, there are three other proteins (Vca1114GL_03262, Vca1114GL_03492, and Vca1114GL_04269) each of which has two MatE domains. Because a protein with one or more MatE domains can function as a drug/sodium antiporter, variation in the copy number of these antiporter proteins among *V. campbellii* strains may cause variation in multidrug resistance strength. In addition, one adhesion-related autotransporter β-domain was present in every member of Vc-Gr1, but absent from Vc-Gr2 (Fig. 3, Supplementary Fig. S1a, and Table S5d). In contrast, 155 domains were significantly expanded in Vc-Gr2 (see Supplementary Fig. S1b and Table S5e) and were enriched in mobile genetic element terms (see Supplementary Table S7b). The galactose metabolic process was originally thought to be absent in *V. campbellii* and this phenotypic feature was one of those used in the traditional diagnosis to distinguish between *V. campbellii* and *V. harveyi*^41^,^42^. However, the genes responsible for D-galactose fermentation are all present in 1114GL, including 1114GL_02654 (Aldose 1-epimerase; EC 5.1.3.3), 1114GL_02653 (galactokinase; EC 2.7.1.6), 1114GL_02652 (UDP-glucose-hexose-1-phosphate uridylyltransferase; EC 2.7.7.12), 1114GL_02651 and 1114GL_04334 (both UTP-glucose 4-epimerase, EC 5.1.3.2), 1114GL_02322 (UTP-glucose-1-phosphate uridylyltransferase, EC 2.7.7.9), and 1114GL_01168 (phosphoglucomutase, EC 5.4.2.2). Additionally, a carbohydrate fermentation test of 1114GL showed acid production when using D-galactose as the sole carbon source (see Supplementary Fig. S2a), and 1114GL can grow better in basal medium (0.2% peptone and 0.1% yeast extract) with D-galactose than in basal medium without D-galactose (see Supplementary Fig. S2b). In *V. campbellii* strains, aldose 1-epimerase,galactokinase, and UDP-glucose-hexose-1-phosphate uridylyltransferase are absent only in NBRC15631 and DS40M4 (see Supplementary Fig. S3). This finding is consistent with a recent suggestion that the utilization of D-galactose is actually a variable feature among *V. campbellii*^22^ strains.

In the Vc-Gr1 enriched domains, the Endotoxin_N domain was present in both KC13.17.5 and 151112 C, but absent in all other strains analyzed in this study. This led us to identify the two strains with a PirB, which is known as a cause for acute hepatopancreatic necrosis disease (AHPND) in shrimps by *V. parahaemolyticus*^43^. Compared with the PirB protein (accession number: AKC05670.1) from *V. parahaemolyticus* 3HP in which the virulence of this protein has been identified, the protein sequence identity of the PirB in KC13.17.5 (WP_025789543.1) is 100% and that in 151112 C (WP_045384430.1) is 70.3%. Although PirB was seen in KC13.17.5 in a previous study^44^, this strain was misidentified as *V. harveyi*. 151112 C containing PirB has never been studied with respect to whether it can cause AHPND or not. Here, we confirmed that the *pir*B gene is present only in Vc-Gr1.

### Expanded domains, especially Big_2, found in *V. campbellii* 1114GL

To identify potential virulent factors of *V. campbellii* 1114GL to shrimps, expanded or reduced Pfam domains in 1114GL were found by comparing *V. campbellii* 1114GL to the 41 other strains (Wilcoxon rank-sum test; p < 0.05). We found 29 domain families significantly expanded in 1114GL (see Supplementary Table S5f), of which 15 displayed a 4-fold or higher average copy number. One interesting domain is Big_2 (22 versus 2.54 copies on average), which modulates bacterial cell-adhesion^45^. Another is the RebB domain (4 versus 0.15), which is responsible for the synthesis of R-body, a complex protein inclusion associated with toxic effects of *Caedibacter* cells on host paramecia^46^,^47^.

**Figure 4:**
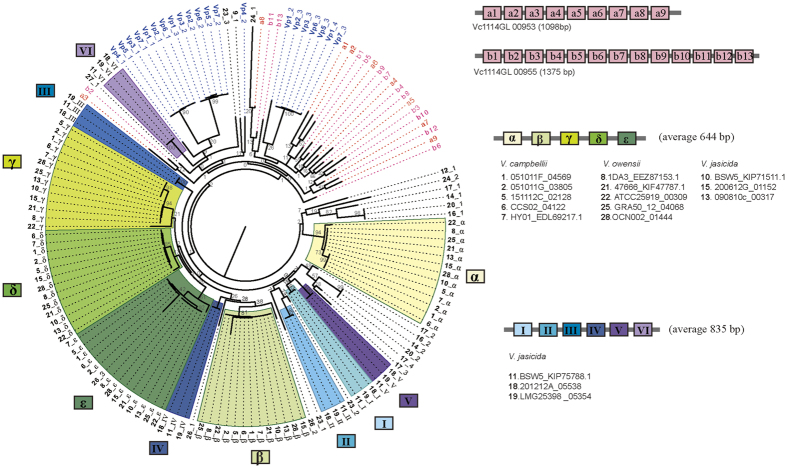
Phylogenetic tree based on Big_2 domains. The Big_2 domains used were 47 Big_2 domains from 6 *V. campbellii* strains, 34 domains from 6 *V. owensii* strains, 44 domains from 7 *V. jasicida* strains, 1 domain from *V. harveyi*, and 21 domains from *V. parahaemolyticus*. The 9 Big_2 domains of gene 00953 and 13 of gene 00955 from 1114GL are highlighted by a1–a9 and b1–b13, respectively. The name of each unit is a domain from a gene (denoted as “gene_domain”). The genes are represented by the following numbers (in bold face): **1–7**: *V. campbellii* 051011 F_04569, 051011G_03805, Vc1114GL_00953, 1114GL_00955, 151112C_02128, CCS02_04122, and HY01_EDL69217.1. **8**: *V. owensii* 1DA3_EEZ87153.1. **9**: *V. harveyi* AOD131_02851. **10–20**: *V. jasicida* BSW5_KIP71511.1, BSW5_KIP75788.1, BSW7_ KIP71411.1, 090810c_00317, 090810c_00949, 200612G_01152, 200612G_02166, 201212A_03844, 201212A_05538, LMG25398_05354, and MWB21_01073. **21–28**: *V. owensii* 47666-1_KIF47787.1, ATCC25919_00309, ATCC25919_01392, ATCC25919_03479, GRA50-12_04068, LMG25430_00078, LMG25430_00181, and OCN002_01444. **Vp1–Vp7**: *V. parahaemolyticus* BB22OP_AGB10101.1, CDCK4557_AGQ98222.1, FDAR31_AGQ91073.1, FDAR31_AGQ94143.1, FORC008_AKU54820.1, RIMD2210633_BAC60030.1, and UCMV493_AHI99806.1.

The Big_2 domain gene was found in tandem within bacterial immunoglobulin-like (Ig-like) genes, and was only observed in 6 Vc-Gr1 strains. Within the core of the Harveyi clade, Ig-like genes were found in ~47.6% of the strains, which on average had 4.6 copies of Big_2 domains. Strikingly, 1114GL contained two Ig-like genes with 9 and 13 Big_2 domains. In the light of the species phylogeny, Ig-like genes seemed to be ancestral to the core Harveyi clade and were subsequently lost, especially in the *V. harveyi* and *V. rotiferianus* groups (Fig. 2a). Interestingly, the phylogeny of *V. campbellii* Big_2 domains revealed strong clustering according to their physical location within Ig-like genes with the exception of those in 1114GL (Fig. 4). All of the 1114GL Big_2 domains were clustered in a clade, suggesting that strong intragenic or intergenic reshuffling among these domains has taken place. Little is known about the function of the Big_2 domain. The best studied case is in *Escherichia coli*: the Big_2 domain is found in the inimin protein, which is the ligand for Tir (Translocated intimin receptor)^45^. Currently, the function of Ig-like genes and the mechanism for the expansion of Big_2 domains in *V. campbellii* 1114GL remains elusive.

### Mobile genetic elements and superintegrons are responsible for syntenic differences between *V. campbellii* 1114GL and ATCC BAA-1116

To reveal the syntenic differences between Vc-Gr1 and Vc-Gr2, we compared *V. campbellii* 1114GL, which belongs to Vc-Gr1, and ATCC BAA-1116, which belongs to Vc-Gr2. Most regions could be aligned between the two strains with an average nucleotide identity of 96% (see Supplementary Fig. S4). The relative numbers and positions of the nine rRNA operons were similar between the two strains, indicating that the assembly quality of 1114GL was comparable to the 454 and Sanger sequenced ATCC BAA-1116 (Accession numbers: NC_009777, NC_009783, and NC_009784). We further identified 95 (87.4% genome coverage) and 99 (85.4%) synteny blocks on ChrI and ChrII of 1114GL, and 96 (79.9%) and 98 (78.6%) blocks on ChrI and ChrII of ATCC BAA-1116 (see Supplementary Table S8). GO enrichment of genes in synteny break regions revealed abundant genetic mobile elements in both strains, especially in ATCC BAA-1116 (see Supplementary Table S9). The number of mobile elements correlated with the size of the break region between syntenies of ATCC BAA-1116 (see Supplementary Fig. S5), suggesting that transposition of these genes contributed to a lower synteny conservation in the genome.

It is known that mobile genetic elements can cause horizontal gene transfer and contribute to adaptation to varying environments and acquisition of virulent traits in *Vibrio*^48^. In an attempt to understand how these transposase genes evolved and led to the differences between ATCC BAA-1116 and 1114GL, a phylogenetic tree was constructed with the transposase sequences of GO:0006313 (transposition, DNA-mediated) from the ten biggest synteny breaks of ATCC BAA-1116 (see Supplementary Fig. S6). The phylogeny revealed two major groups with one having a much higher intra-group distance than the other (66.9% versus 1.3%). The distance between the two groups was 78.4%. We hypothesized that the longer syntenic break regions were a result of preferential transposition of mobile elements to already inserted regions^49^. Indeed, the phylogeny of these proteins showed no correlation in terms of amino acid distance between their relative orders inside syntenic break regions or their relative chromosome positions, suggesting that transpositions across the genome have been largely random but steadily accumulated in regions where syntenies were already broken up.

*V. campbellii* 1114GL harbors a 73 kb super integron (SI) on chromosome I (1114GL_00040 -1114GL_00134; see Supplementary Fig. S7a). This SI has several interesting features. First, its *IntI*4 is identical to the *IntI*4 from *V. harveyi* CAIM 1792 (accession number EMR38874) and has 65.3% sequence identity with the *IntI*4 from *V. cholerae* (accession number NP_232687.1). Second, we detected 39 conserved *att*C sites (see Supplementary Fig. S7b). These *att*C sites are 120 bp long and have 78.3–100% identity with the consensus sequence. Third, 76% of the genes are hypothetical proteins, similar to that in the SI of the other *Vibrio* genomes^50^. Although this SI has no syntenic relationship with *V. campbellii* ATCC BAA-1116, a remnant full-length *IntI*4 was identified on chromosome I.

### Strong purifying selection on *Vibrio campbellii* genes

**Figure 5:**
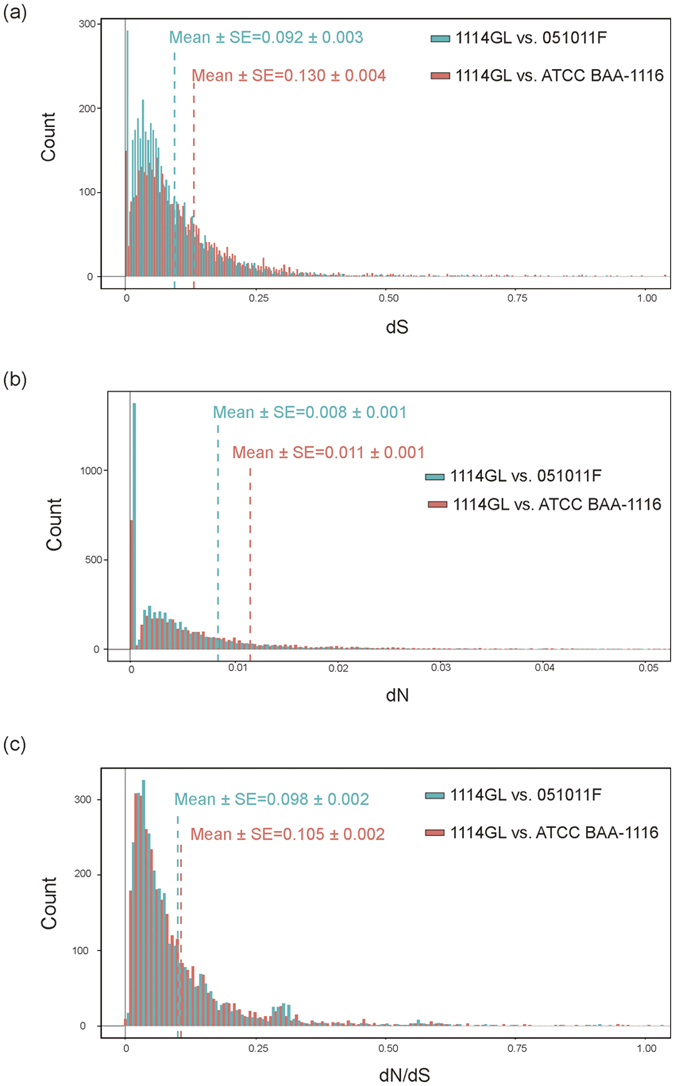
Distribution of sequence divergence between orthologous gene pairs. The dS (**a**), dN (**b**), and dN/dS (**c**) values were calculated from the genes in the syntenic regions between 1114GL and 051011F (Green) and between 1114GL and ATCC BAA-1116 (Red). The y-axis is the number of gene pairs.

We next examined the degree of sequence divergence between *V. campbellii* strains and asked whether positive selection has occurred in any of the genes. dN (number of non-synonymous substitutions per site) and dS (the number of synonymous substitutions per site) were calculated for 4,239 one-to-one orthologous pairs in the syntenic regions between 1114GL and 051011F and for 3,672 pairs in the syntenic regions between 1114GL and ATCC BAA-1116 (Fig. 5). The mean dS value was 0.092 between 1114GL and 051011F and 0.130 between 1114GL and ATCC BAA-1116, while the mean dN values were 0.008 and 0.011, respectively. At a mean dS of 0.092, 051011F was the closest to 1114GL among the strains studied, indicating high genetic diversity of the *V. campbellii* species. The mean dN/dS value of gene pairs was 0.0981 between 1114GL and 051011F and 0.104 between 1114GL and ATCC BAA-1116 (Fig. 5). About 80% of the dN/dS values both between 1114GL and 051011F and between 1114GL and ATCC BAA-1116 were much smaller than 1, a result indicative of strong purifying selection on the majority of the genes in the genome. Moreover, there was no evidence of positive selection for any gene (Fig. 5). This predominant mode of purifying selection was also observed in the study of other prokaryotic genomes^51^.

## Conclusions

In this study, we sequenced, assembled, and annotated the noncontiguous finished genome^52^ of a *Vibrio* isolate (*V. campbellii* 1114GL) that causes shrimp disease in Thailand. We compared this genome to 47 other sequenced *Vibrio* genomes in the core of the Harveyi clade. We showed that the species classification and phylogenetic relationships of the core species in the Harveyi clade could be correctly delineated based on a combination of average nucleotide identity and phylogenetic reconstruction of concatenated single copy orthologous genes. This enabled us to carry out an extensive comparative analysis of the *Vibrio* genomes to reveal how these organisms adapted to different environments. For example, the existence of iron acquisition genes in *V. campbellii* and the expansion of genes associated with transposition in Vc-Gr2 were discovered. This study demonstrated that a high quality genome assembly can provide more accurate gene annotation and taxonomic classification, and can enable detailed analyses including synteny analysis. We were thus able to describe the evolutionary dynamics at the genome level, such as strong purifying selection across the genome and numerous genomic rearrangements caused by transposition of mobile genetic elements. Areas worth further investigation have also been highlighted. First, the roles of Ig-like genes in different *Vibrio* species warrant functional studies. Second, it will be interesting to investigate whether the PirB protein in 151112 C can cause acute hepatopancreatic necrosis disease as in *V. campbellii* strain KC13.17.5. Finally, further comparative genomic analyses of the Harveyi clade members will provide deeper biological insights.

## Materials and Methods

### DNA preparation, sequencing and *de novo* assembly of the *V. campbellii* 1114GL genome

The *V. campbellii* 1114GL strain, provided by Timothy William Flegel, Centex Shrimp, Faculty of Science, Mahidol University, Bangkok, Thailand, was named VH1114GL in a previous study^28^. It was cultured from a glycerol stock in MHB + 3% ASW at 30 °C with shaking at 200 rpm for 16 hours. The bacterial cells were pelleted by centrifugation at 5,000 × g for 10 min. Genomic DNA was extracted using Qiagen Genomic-tip 100/G according to the manufacturer’s instructions. For genome sequencing, Illumina and Roche 454 platforms were used. Two paired-end libraries (insert size = ~320 bp) were constructed using the TruSeq DNA Preparation Kit with the standard protocol (Illumina) and sequenced by Illumina MiSeq to produce 250-bp paired end reads. Three mate-pair libraries of various jumping sizes (2 kb, 4 kb, and 6 kb) were constructed using the Nextera Mate Pair Sample Preparation Kit and sequenced by Illumina HiSeq2000 to produce 100-bp mate pair reads. The long single-end reads were produced by 454 GS FLX+.

### Genomic data

Before the assembly of the 1114GL genome, adaptor and quality trimming (Q30 with minimum length = 70 bp) were conducted using Trimmomatic (version 0.32)^53^ leaving 4,968,772 paired-end sequences were retained. A total of 3,786,218 mate-pair sequence reads were retained after the detection and quality-trimming of TruSeq adaptor with at least 50 bp reserved in both reads using Nextclip^54^. The paired-end, mate-pair, and raw 454 reads (437,632 reads with mean length = 980 bp) of 1114GL were assembled *de novo* using the ALLPATH-LG assembler (ver. r48123)^55^. Subsequently, gap were closed using GapFiller (v1-10)^56^. Another 64 *Vibrio* genome assemblies were downloaded from NCBI (last retrieval date: 18^th^ September 2015; see Supplementary Table S1).

For taxonomy confirmation, the Average Nucleotide Identity (ANI) for which pair-wise comparison of sequences between two strains or species was calculated using pyani (https://github.com/widdowquinn/pyani/tree/master/pyani) with BLAST method^23^.

### Predicted proteins

The *V. campbellii* 1114GL assembly and the publicly available assemblies without annotation were annotated by the Prokaryotic Genome Annotation System (PROKKA) pipeline^29^. Functional annotation of the predicted proteins was obtained by Argot2^57^. Protein domains of each gene were identified by pfam_scan.pl v1.5 by comparing against Pfam v27.0^58^. The replication origin (*oriC*) regions of bacterial genomes were predicted by the online system Ori-Finder^59^ and DoriC^60^ tools.

### Phylogenetic analysis

Single-copy orthologous genes in all 65 *Vibrio* strains of the core Harveyi members (21 from *Vibrio campbellii,* 24 from *V. harveyi,* 7 from *V. owensii,* 5 from *V. jasicida,* 3 *V. rotiferianus*, and 6 from *V. parahaemolyticus*) (Supplementary Table S1) were collected and identified based on the BLASTP results (E-value ≤ 10^−5^) using OrthoMCL^40^. To identify the relationship between the number of orthologous genes and assembly contiguity, the correlation between N90 of the genome assembly and number of orthologous genes were calculated (Fig. 1). N90 = 10 kb was chosen as the cutoff. The final dataset consisted of 48 strains (12 from *V. campbellii* (including 1114GL), 15 from *V. harveyi,* 7 from *V. owensii,* 5 from *V. jasicida,* 3 *V. rotiferianus*, and 6 from *V. parahaemolyticus*) (see Supplementary Fig. S1) with 1,729 single copy orthologs retained after removing alignments with more than 10% gaps of the 1,823 single copy orthologs. A phylogenetic tree was generated using RAxML (v8.1.17)^61^ with 500 bootstrap replicates from the alignment of these concatenated single copy orthologs by MAFFT (v7.123b, local option)^62^. The phylogeny was plotted using FigTree v1.4.2 (http://tree.bio.ed.ac.uk/software/figtree/).

For the phylogenetic tree of specific proteins such as Big_2 (Bacterial Ig-like, group 2) domains and transposase, the corresponding protein sequences were aligned by MAFFT (v7.123b, option localpair)^57^ and trimmed by TrimAl (with option strictplus)^63^. Maximum likelihood phylogenies were constructed by RAxML (v8.1.17)^61^ with 500 bootstraps.

### Identification of genes for classification of the core Harveyi clade and *V. campbellii* groups

To identify the genes informative for taxonomic classification, we develop a method based on the calculation of correlations between the branch length of the Harveyi clade phylogenetic tree and sequence similarities of single copy orthologues. Our procedure includes three steps. First, for a given phylogenic tree with N nodes (species or strains), a distance vector (*V*) was constructed by 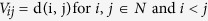, where *i* and *j* were two species in the tree and d(i, j) was the summation of branch lengths between the two species. Second, for a set of orthologous genes (1-to-1 orthologous pairs among N species/strains), a vector of sequence dissimilarity (*S*) between the orthologous pairs was constructed by 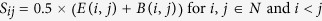. For *E(i, j*), we took log_e_ of the BLAST E-value between the orthologous pair in species *i* and *j*. If the E-value was zero, we set it to 0.1×(minimum non-zero E-value). The log-Evalue (e) was normalized by (e-Emin)/(Emax-Emin), where Emax and Emin were the maximum and minimum log-Evalue, respectively. For *B(i, j*), we normalized the bit score (bs) between the orthologous pair in species *i* and *j* by (bs-bs_max)/(bs_min-bs_max), where bs_max and bs_min were the maximum and minimum of the bit scores, respectively. We transformed the maximum bit score into zero to indicate the lowest sequence dissimilarity. Finally, we calculated Pearson Correlation Coefficient (PCC) between the distance vector (*V*) of the tree and the sequence vector (*S*) for each set of orthologous groups. The PCCs were then sorted in descending order. To obtain the granularity of taxon-specific genes in *Vibrio species* and *V. campbellii* strains, we used two phylogenies: (1) the tree of the *Vibrio* core Harveyi clade with *V. parahaemolyticus* as the outgroup and (2) its sub-tree in the *V. campbellii* species.

Genes identified by this method were aligned by MAFFT (v7.123b, option localpair) and the maximum likelihood phylogenetic tree was constructed by RAxML (v8.1.17) with 500 bootstraps.

### Expansion and reduction of protein domain family sizes

Enrichment of Pfam domain number between two sets of interest was assessed by the Wilcoxon rank-sum test (p ≤ 0.05). We compared the Pfam copy number (1) between *V. campbellii* 1114GL and strains of all others species in the core Harveyi clade, (2) between groups 1 and 2 of *V. campbellii* strains, and (3) between *V. campbellii* and all other species in the core Harveyi clade. The expanded or reduced domains were assigned to the KEGG pathway by KAAS^64^. GO enrichments were identified for significant domain gains/losses using TopGO (version 2.10.0)^65^.

### Synteny analysis

The Artemis Comparison Tool (ACT)^66^ was used to visualize whole-genome alignment between *V. campbellii* 1114GL and ATCC BAA-1116. Synteny block and orthologous gene pairs between 1114GL and ATCC BAA-1116 were defined using DAGCHAINER (-Z 12 -D 3 -g 1 -A 3)^67^, using the BLASTP output with an E-value < 1 × 10^−10^. The sequence regions not covered by synteny blocks were defined as synteny breaks. The genes located in synteny blocks or breaks were assessed by BEDTools^68^ and custom Python scripts.

Single orthologues between pairs of species in synteny blocks were obtained from DAGCHAINER^62^. Genes with one-to-many or many-to-many hits between two genomes were excluded in our sequence divergence analysis. There were 3,672 and 4,239 one-to-one orthologous pairs for 1114GL vs. ATCC BAA-1116 and for 1114GL vs. 051011 F, respectively. For each orthologous pair, dN (number of non-synonymous substitutions per site), dS (the number of synonymous substitutions per site), and dN/dS were computed using the Nei-Gojobori method^69^, and the likelihood values of dN/dS for gene pairs were calculated by PAML^70^. The likelihood ratio test was conducted to test if the dN/dS ratio was significantly different from 1.

### Identification of integrons in *V. cambellii* 1114GL and ATCC BAA-1116

The complete proteome of *V. campbellii* 1114GL and ATCC BAA-1116 were searched against the IntI4 protein sequence of *V. harveyi CAIM 1792* (Genbank accession EMR38874) and *V. cholerae* (Genabank accession NP_232687.1) using blastp. Conserved palindrome sequences were searched from 10 bp before the stop codon to 3′ intergenic sequences at 10 genes downstream of IntI4 using *palindrome* from the EMBOSS package^71^. A final conserved palindrome (TAACNNN[C/T]TGTTNAAG) was used to identify putative cassette-associated recombination (*att*C) site for all intergenic regions in the genomes of 1114GL and BAA-1116. Integron_finder^72^ was also used to check the accuracy of our approach. Multiple alignment of *att*C sites was manually checked using Jalview^73^.

### D-galactose utilization test

1114GL was cultured from a glycerol stock in MHB + 3% ASW at 30 °C with shaking at 200 rpm for 16 hours. After the overnight culture, cells were inoculated, in three replicates, at a starting density of OD_600_ = 0.05 into flasks with 50 ml basal medium (0.2% of peptone and 0.1% of yeast extract broth) and into flasks with 50 ml basal medium plus 0.5% D-galactose. The cells were cultured at 30 °C with shaking at 200 rpm. Their growth was monitored by measuring the OD_600_ every hour.

For the D-galactose fermentation test, the overnight culture was inoculated into 4 ml basal media at a starting density of OD_600_ = 0.05 with and without 1.8 × 10^−3^% of phenol red. After cultures were grown at 30 °C for 10 hours, the culture color was assessed. A change in color from red to yellow indicates acid production.

## Additional Information

**How to cite this article:** Ke, H. M. *et al*. Comparative genomics of *Vibrio campbellii* strains and core species of the *Vibrio* Harveyi clade. *Sci. Rep.* **7**, 41394; doi: 10.1038/srep41394 (2017).

**Publisher's note:** Springer Nature remains neutral with regard to jurisdictional claims in published maps and institutional affiliations.

## Supplementary Material

Supplementary Figures

Supplementary Tables S1-S9
