# Supplementary material for: Comparative genomics of Vibrio campbellii strains and core species of the Vibrio Harveyi clade
Source: Sci Rep. 2017 Feb 1;7:41394. doi: 10.1038/srep41394 (PMC5286417; doi:10.1038/srep41394)

Comparative genomics of *Vibrio campbellii* strains and core species of the *Vibrio* Harveyi clade

Huei-Mien Ke[1,2], Anuphap Prachumwat[3,4], Chun-Ping Yu[2], Yi-Ting Yang[5,6], Sutitcha Promsri[3,7], Kuan-Fu Liu[8], Chu-Fang Lo[5,6,9], Mei-Yeh Jade Lu[2], Mei-Chin Lai[1,10,11], Isheng J Tsai[1,2,*], and Wen-Hsiung Li[1,2,12,*]

1 Ph.D. Program in Microbial Genomics, National Chung Hsing University and Academia Sinica, Taiwan.
2 Biodiversity Research Center, Academia Sinica, Taipei, Taiwan.
3 Centex Shrimp, Faculty of Science, Mahidol University, Bangkok, Thailand.
4 Shrimp-Virus Interaction Laboratory, Animal Biotechnology Research Unit, National Center for Genetic Engineering and Biotechnology (BIOTEC), National Science and Technology Development Agency (NSTDA), Pathum Thani, Thailand.
5 Institute of Bioinformatics and Biosignal Transduction, College of Bioscience and Biotechnology, National Cheng Kung University, Tainan, Taiwan.
6 Department of Life Science, National Taiwan University, Taipei, Taiwan.
7 Department of Biotechnology, Faculty of Science, Mahidol University, Bangkok, Thailand.
8 Tungkang Biotechnology Research Center, Fisheries Research Institute, Council of Agriculture, Pingtung, Taiwan.
9 Center of Bioscience and Biotechnology, National Cheng Kung University, Tainan, Taiwan.
10 Department of Life Sciences, National Chung Hsing University, Taichung, Taiwan.
11 Agricultural Biotechnology Center, National Chung Hsing University, Taichung, Taiwan.
12 Department of Ecology and Evolution, University of Chicago, Chicago, US.

*Author for Correspondence: Isheng J Tsai, Biodiversity Research Center, Academia Sinica, Taipei, Taiwan, ijtsai@gate.sinica.edu.tw, and Wen-Hsiung Li, Biodiversity Research Center, Academia Sinica, Taipei, Taiwan, whli@uchicago.edu.

Figure S1. Protein domain enrichment across strains. Protein domains expanded (Wilcoxon rank sum test, $p < 0.05$; 4-fold copy number) either in Group 1 (S1a) or Group 2 (S1b) were colored from light to dark blue and the number is rescaled between 0 to 1 (for each domain, copy number from each of 48 strains is divided by the maximum copy number).
The names of protein domains are labeled in the horizontal axis and the names of strains are labeled in the vertical axis.
The domain of unknown function (DUF) is not shown.

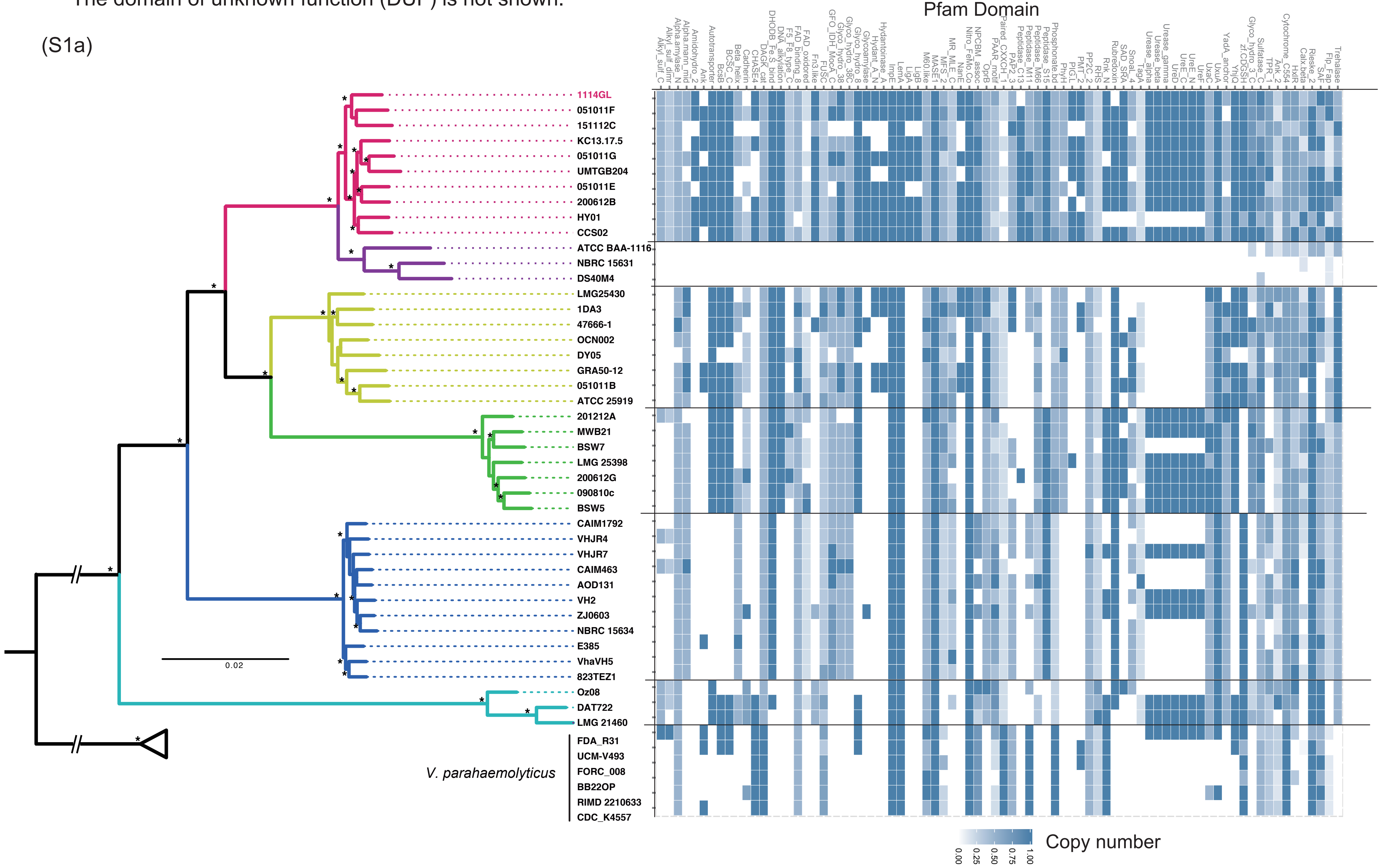

(S1b)

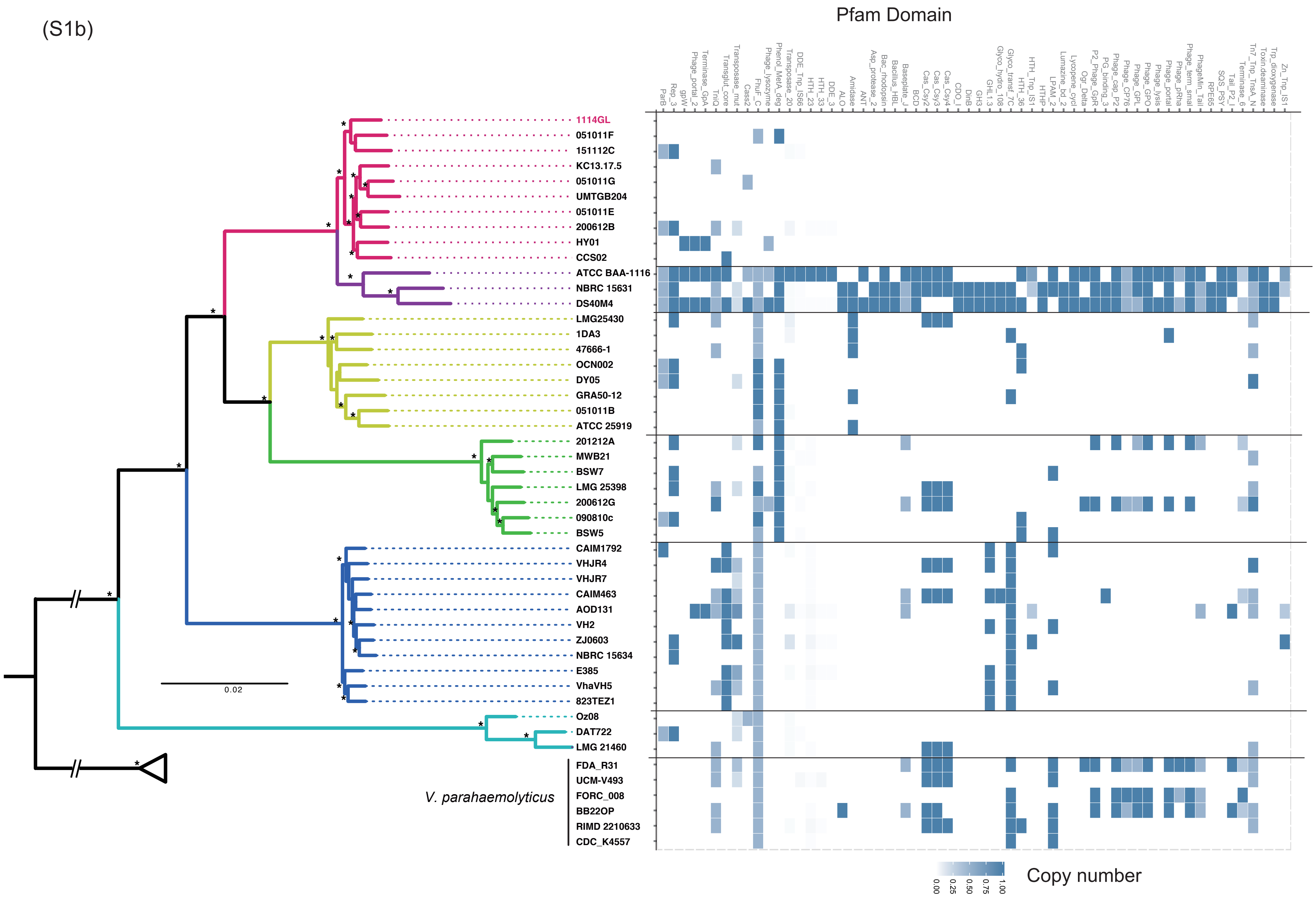

Pfam Domain
ParB
Rep_3
gpW
Phage_portal_2
Terminase_GpA
TniQ
Transglut_core
Transposase_mut
Cass2
FhuF_C
Phage_lysozyme
Phenol_MetA_deg
Transposase_20
DDE_Tnp_IS66
HTH_23
HTH_33
DDE_3
ALO
Amidase
ANT
Asp_protease_2
Bac_rhodopsin
Bacillus_HBL
Baseplate_J
BCD
Cas_Csy2
Cas_Csy3
Cas_Csy4
CDO_I
DinB
GH3
GHL1-3
Glyco_hydro_108
Glyco_transf_7C
HTH_36
HTH_Tnp_IS1
HTHP
LPAM_2
Lumazine_bd_2
Lycopene_cycl
Ogr_Delta
P2_Phage_GpR
PG_binding_3
Phage_cap_P2
Phage_CP76
Phage_GPL
Phage_GPO
Phage_lysis
Phage_portal
Phage_pRha
Phage_term_smal
PhageMin_Tail
RPE65
SQS_PSY
Tail_P2_I
Terminase_6
Tn7_Tnp_TnsA_N
Toxin_deaminase
Trp_dioxygenase
Zn_Tnp_IS1
1114GL
051011F
151112C
KC13.17.5
051011G
UMTGB204
051011E
200612B
HY01
CCS02
ATCC BAA-1116
NBRC 15631
DS40M4
LMG25430
1DA3
47666-1
OCN002
DY05
GRA50-12
051011B
ATCC 25919
201212A
MWB21
BSW7
LMG 25398
200612G
090810c
BSW5
CAIM1792
VHJR4
VHJR7
CAIM463
AOD131
VH2
ZJ0603
NBRC 15634
E385
VhaVH5
823TEZ1
Oz08
DAT722
LMG 21460
FDA_R31
UCM-V493
FORC_008
BB22OP
RIMD 2210633
CDC_K4557
V. parahaemolyticus
0.02
Copy number
0.00
0.25
0.50
0.75
1.00

Figure S2a. The carbohydrate fermentation test of 1114GL showed acid production when using D-galactose as the sole carbon source.
Tube 1: basal medium without D-galactose and without 1114GL;
Tubes 2-4: basal medium without D-galactose but inoculated with 1114GL;
Tube 5: basal medium with D-galactose but without 1114GL;
and Tubes 6-8: basal medium with D-galactose but inoculated with 1114GL.
The color was observed after 10 hours.

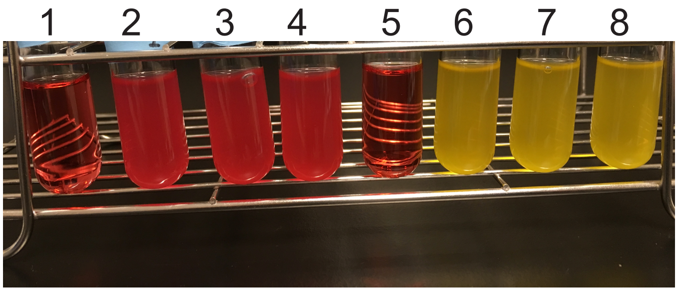

Figure S2b. The growth curves of 1114GL on basal medium and on basal medium with D-galactose. Three replicates were conducted for both conditions.

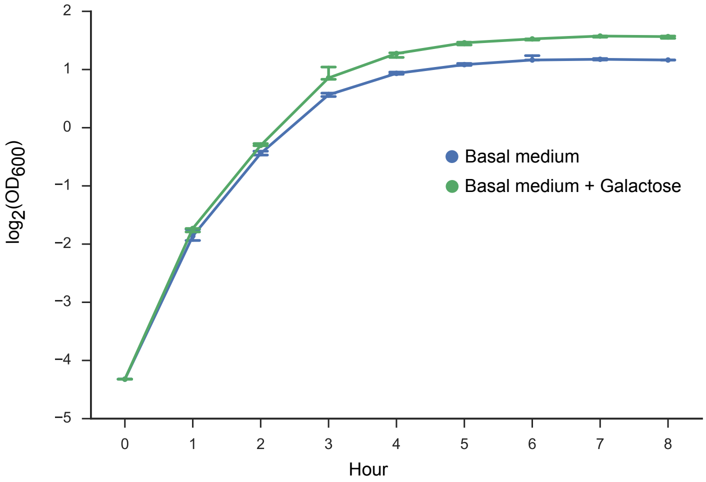

Figure S3. The number of the proteins related to galactose fermentation in *V. campbellii*.

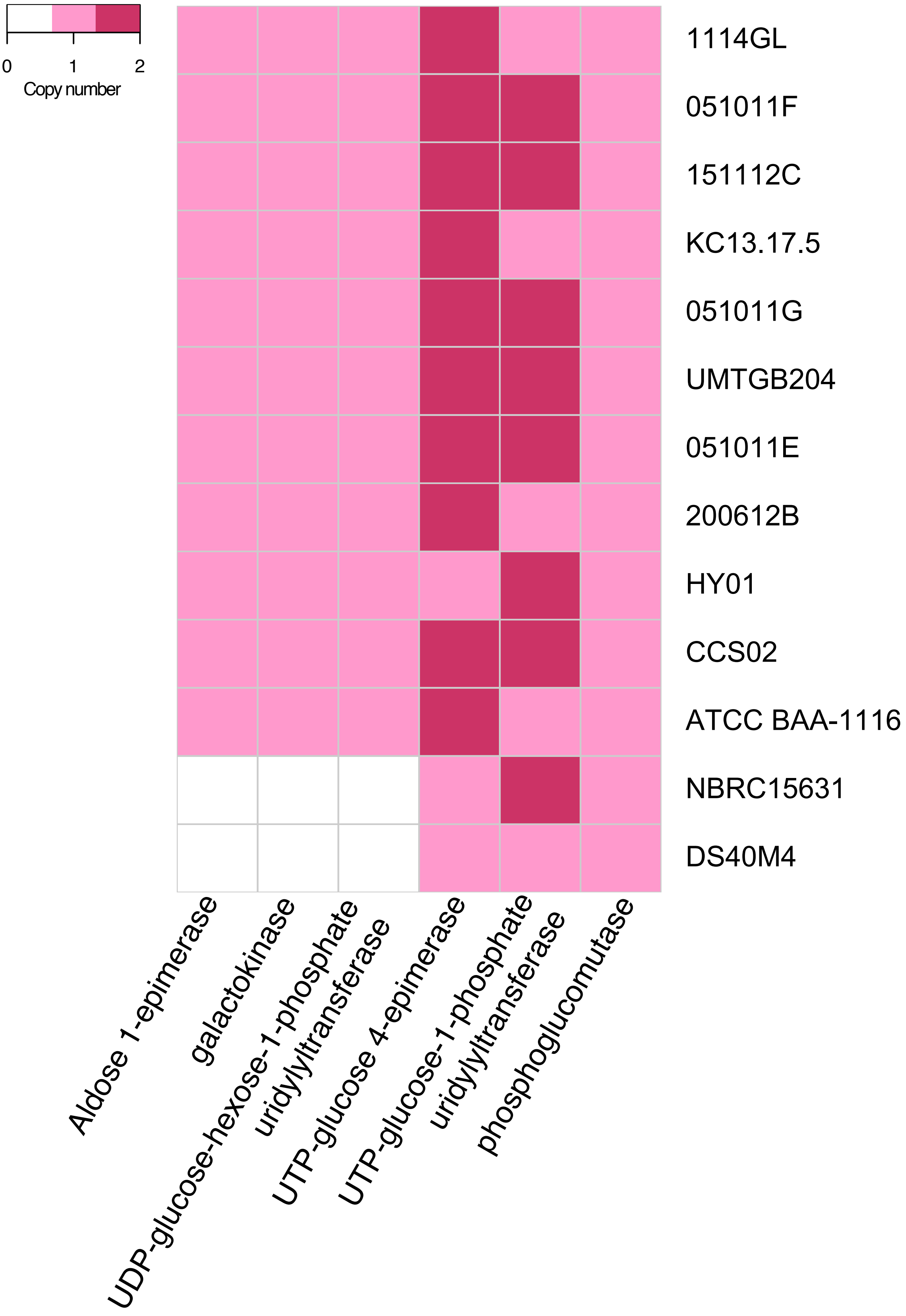

Figure S4. The sequence alignment of Chromosome I (a) and Chromosome II (b) between V. campbellii 1114GL and ATCC BAA-1116.

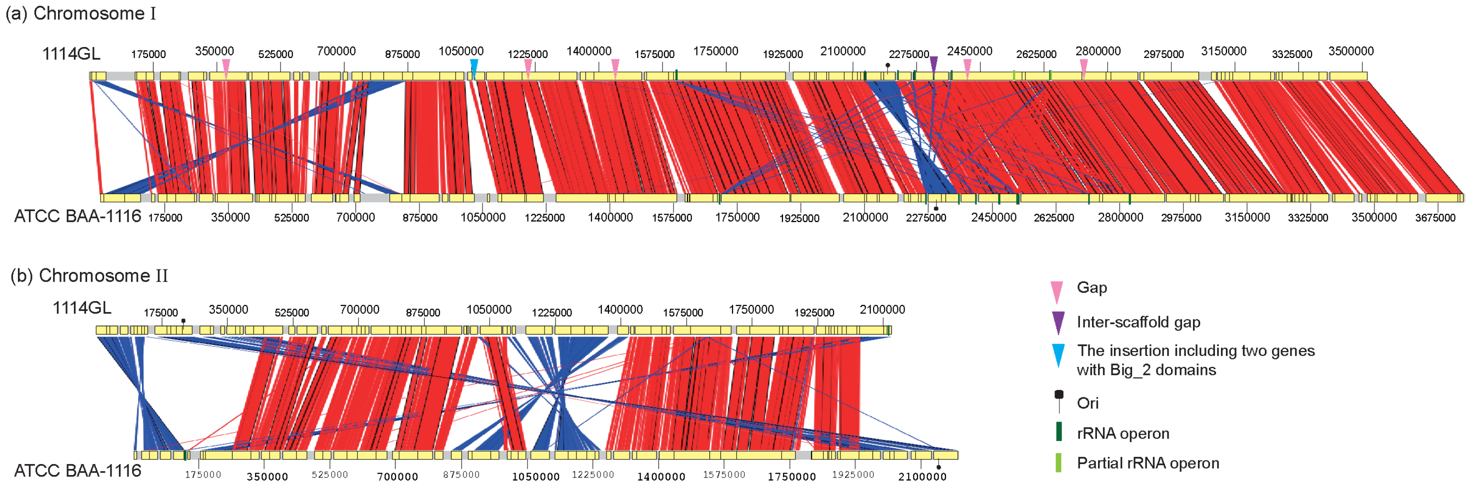

Figure S5. Correlation between transposase gene number and the size of synteny break.

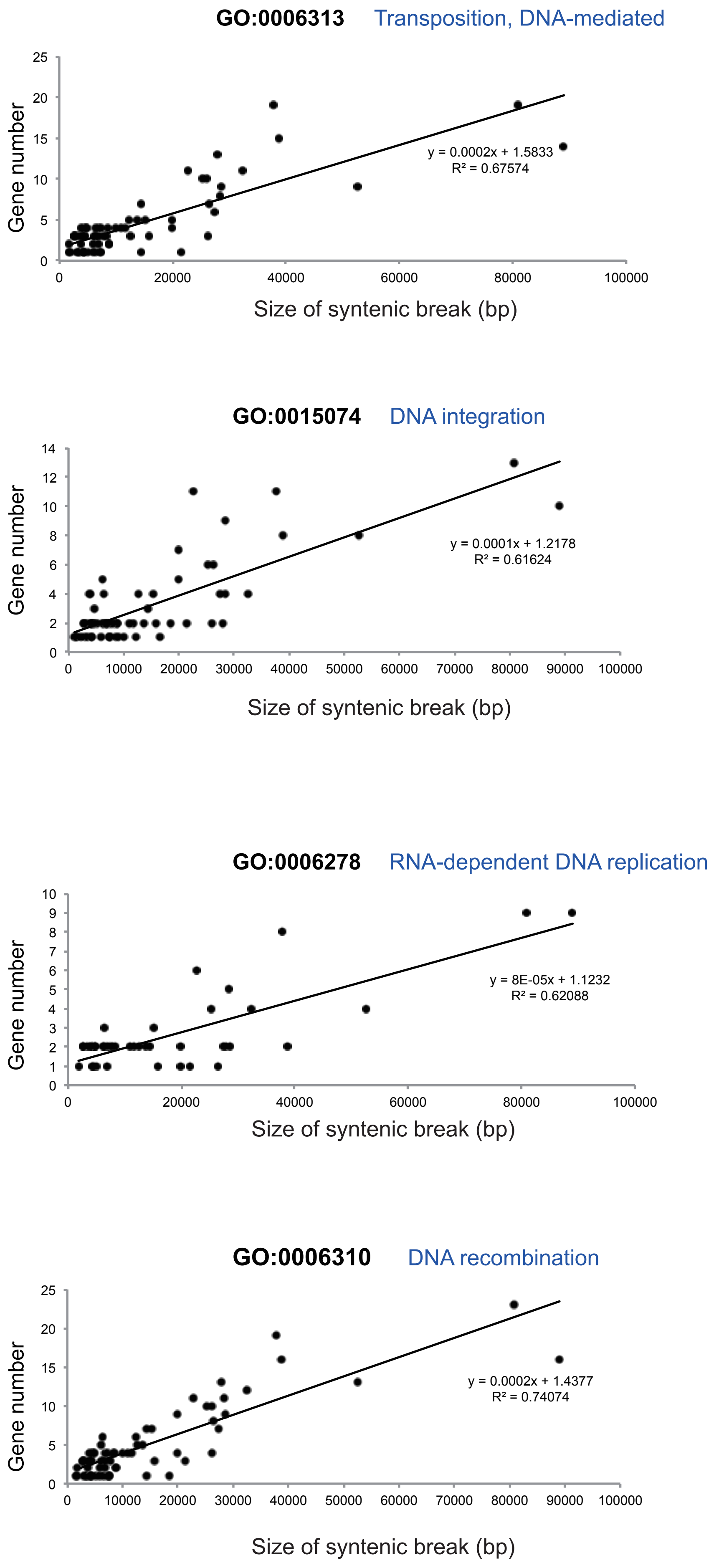

Figure S6. Phylogenetic tree based on the transposase protein sequences from the synteny break.
The color of a lineage is labeled according to its position in the synteny break.

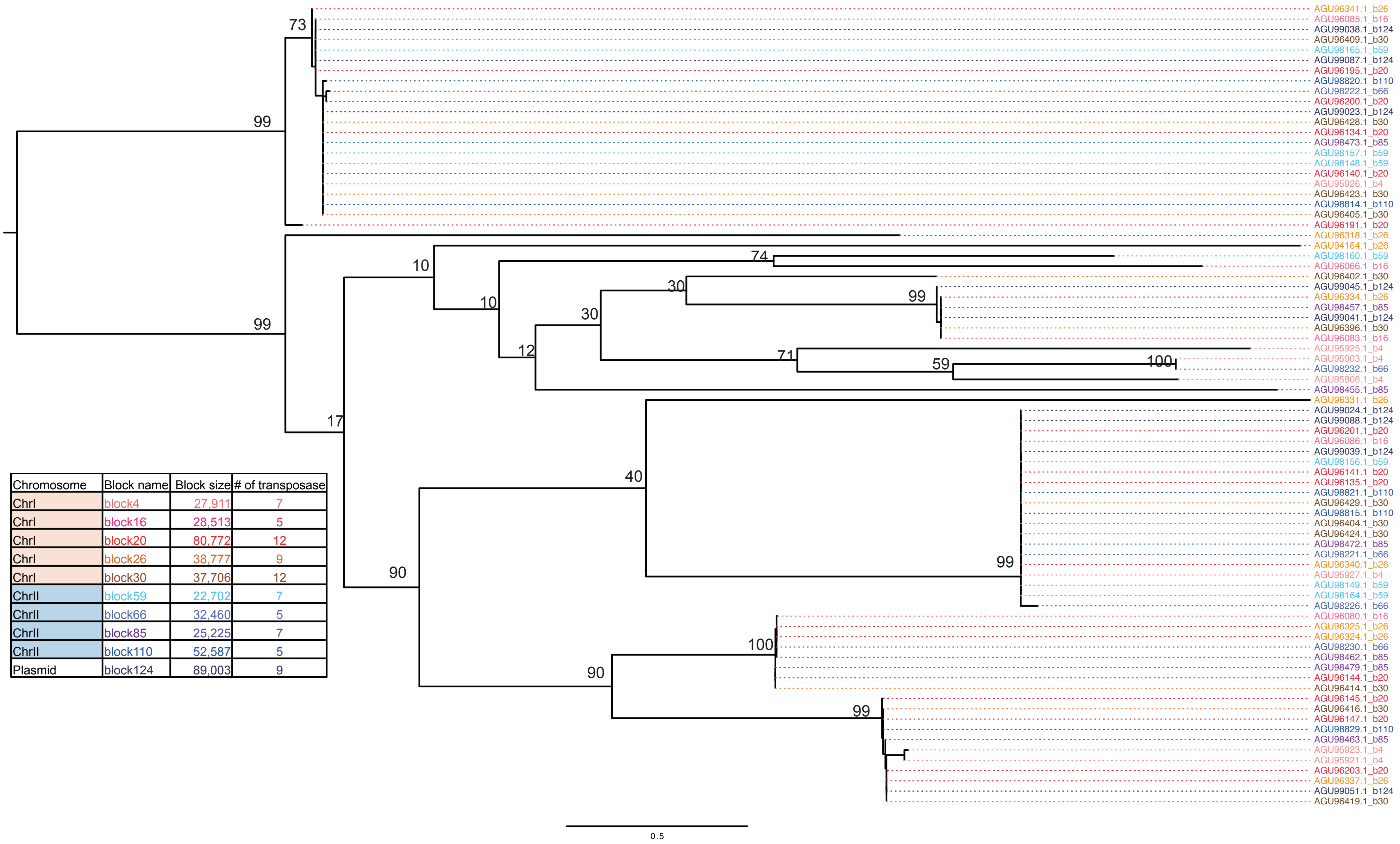

Chromosome	Block name	Block size	# of transposase
ChrI	block4	27,911	7
ChrI	block16	28,513	5
ChrI	block20	80,772	12
ChrI	block26	38,777	9
ChrI	block30	37,706	12
ChrII	block59	22,702	7
ChrII	block66	32,460	5
ChrII	block85	25,225	7
ChrII	block110	52,587	5
Plasmid	block124	89,003	9

Figure S7a. Diagram of V. campbellii 1114GL superintegron gene cassette array. 39 aatC sites were pred
across the coding sequence from Vca1114GL_00040 to Vca1114GL_00146 (box arrow).

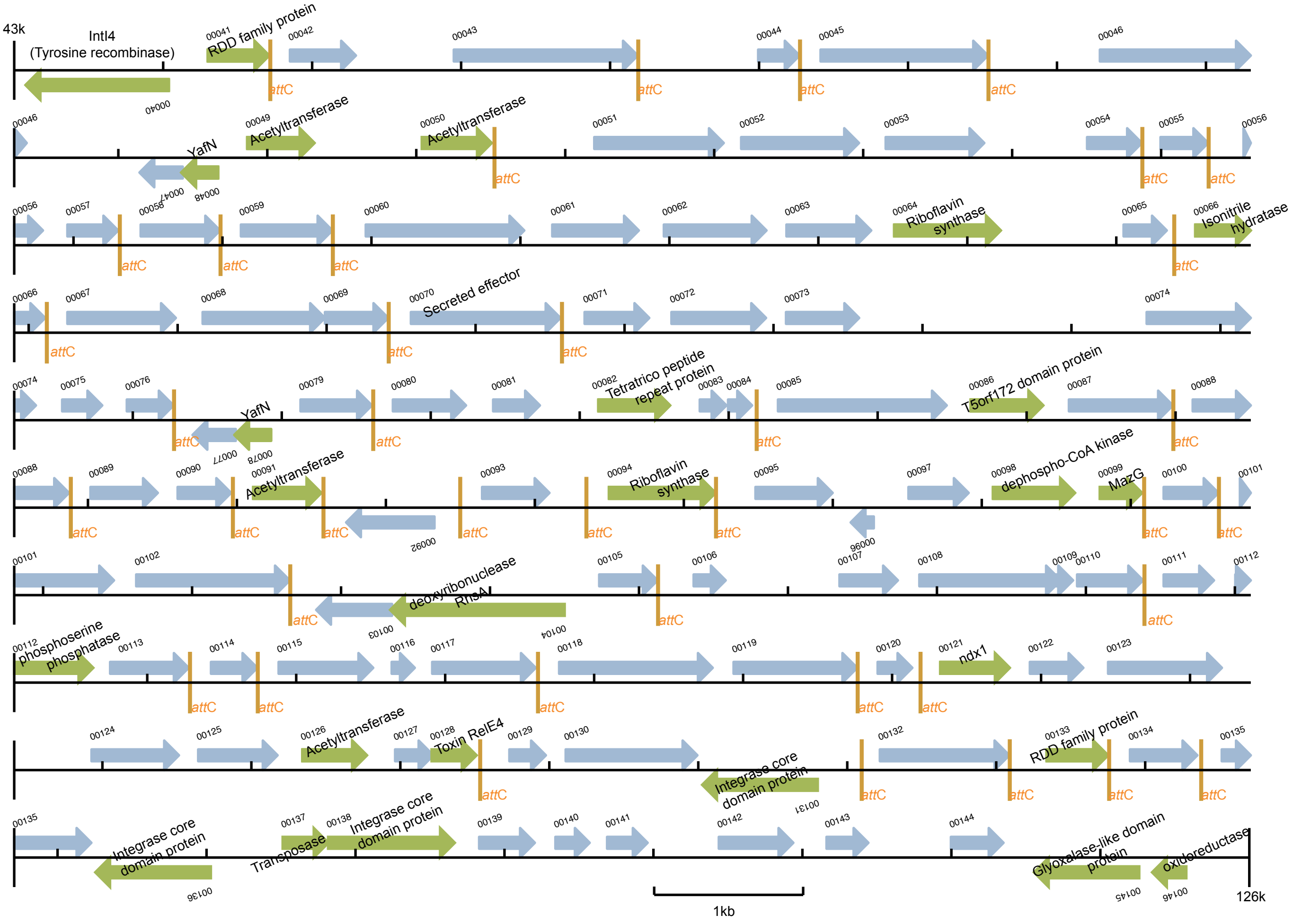

Figure S7b. The alignment of 39 predicted attC site containing partially palindromic sequences (labeled with R’, R”, L’, and L”).

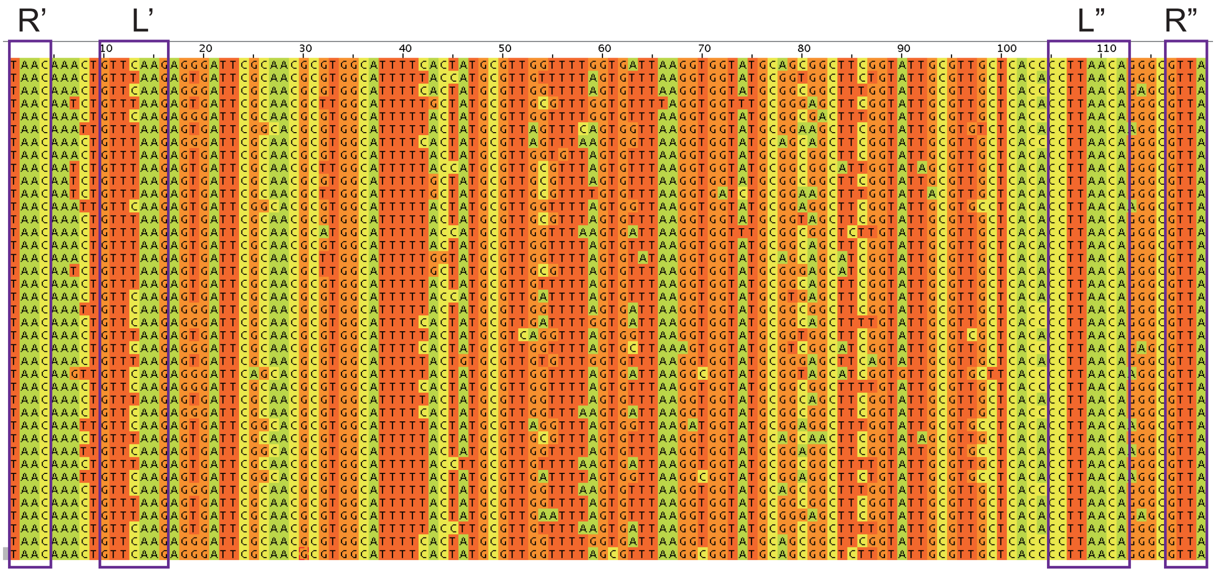

Supplement: Supplementary Figures [file srep41394-s1.pdf]
